# Effect of non-surgical periodontal therapy on CD14 + CD16+ monocyte counts in peripheral blood samples: a clinical interventional study

**DOI:** 10.1186/s12903-023-03793-3

**Published:** 2024-01-17

**Authors:** Raghunanthan Jagannathan, Thodur Madapusi Balaji, Suresh Ranga Rao, Meshal Saleh Alosaimi, Shankargouda Patil, Yuliia Siurkel, Marco Cicciù, Giuseppe Minervini

**Affiliations:** 1 Department of Periodontics and Implantology, Faculty of Dental Sciences, Sri Ramchandra University, Chennai, 600116 India; 2 Tagore Dental College and Hospital, Chennai, Tamilnadu 600127 India; 3 Department of Periodontics and Implantology, Faculty of Dental Sciences, Sri Ramchandra University, Chennai, 600116 India; 4 Dental hospital, King Saud University, Riyadh, 145111 Saudi Arabia; 5 College of Dental Medicine, Roseman University of Health Sciences, South Jordan, UTAH 84095 USA; 6 International European University School of Medicine, AkademikaHlushkova Ave, 42В, Kyiv, 03187 Ukraine; 7 Department of General Surgery and Surgical-Medical Specialties, School of Dentistry, University of Catania, Catania, 95124 Italy; 8 Saveetha Dental College and Hospitals, Saveetha Institute of Medical and Technical Sciences (SIMATS), Saveetha University, Chennai, Tamil Nadu India; 9 Multidisciplinary Department of Medical-Surgical and Dental Specialties, University of Campania Luigi Vanvitelli, Caserta, 81100 Italy

**Keywords:** Chronic periodontitis, IgG, Monocytes, Receptor, Root planing, Scaling

## Abstract

Monocytes and their macrophage progeny are thought to be involved in tissue and alveolar bone destruction in periodontal disease. It has been documented that the proportion of (CD14 + CD16+) non-classical monocytes in the blood are elevated in chronic periodontitis;A total of 20 chronic generalized periodontitis patients who were otherwise healthy, were recruited for this study. At baseline and 3 weeks after non-surgical periodontal treatment, peripheral blood was obtained to assess the levels of C-reactive protein (CRP) and the proportion of monocyte subsets. Monocyte subsets were assessed using flow cytometry;The mean percentage of CD14 + CD16+ non-classical monocytes in the peripheral blood sample at baseline was 13.95 + 2.09, that reduced to 8.94 + 1.23 3 weeks after non-surgical treatment. A distinct significant reduction in the percentage of non-classical monocytes and a concomitant increase in classical monocytes were observed following periodontal treatment compared to baseline. There was a significant reduction in the all the periodontal parameters and CRP levels 3 weeks post non-surgical periodontal treatment. A positive correlation between CRP and percentage of non-classical monocytes was also observed; Periodontal treatment potentially modulates the host response effectively.

## Introduction

Mononuclear cells are primary phagocytes that are highly effective against invading microorganisms. Monocytes and their macrophage progeny are thought to be involved in tissue and alveolar bone destruction in periodontal disease [1]. Chronic periodontitis is an infectious disease characterized by inflammation of the tooth-supporting structures of the teeth [2]. This destruction involves the production of pro-inflammatory cytokines such as IL-1β, TNF and release of matrix metalloproteinases by monocytes and macrophages [3–5]. Monocytes are a heterogeneous population of immune cells that exist in three subsets that can be identified by flow cytometry due to the differential expression of receptors CD14 (co-receptor for toll-like receptor 4) and CD16(Fc gamma receptor IIIa) [6]. The three subsets of monocyte are phenotypically and functionally diverse [7]. The classical monocytes express only CD 14 receptors and comprise of 80–95% of circulating monocytes whereas the non-classical monocytes co-express CD14 and CD16 and constitute 2–11% [8, 9]. These non-classical monocytes are known to possess proinflammatory character and secrete more inflammatory mediators in response to infection [10] and also tend to retain their proinflammatory nature [11]. Other than elaboration of cytokines such as IL-1β, TNF alpha and an array of proteolytic enzymes involved in matrix degradation, these cells also produce certain important acute phase proteins such as C reactive protein (CRP). The elevation of CRP in blood samples, tissue homogenates and Gingival crevicular fluid (GCF) samples of patients with periodontal disease and its reduction following periodontal therapy has been previously documented. The CD14 + CD16+ non-classical subset of monocytes have also been found to be elevated in individuals with chronic periodontitis [12, 13]. It has been documented that the proportion of non-classical monocytes in blood is elevated in chronic periodontitis but it is currently unknown whether a therapeutic intervention can reduce these elevated cells. In the present clinical interventional study we assessed the proportion of monocyte subsets and CRP levels in peripheral blood samples of chronic generalized periodontitis patients at baseline and 3 weeks post non-surgical periodontal therapy to assess if periodontal treatment modulates the proportion of these immune cells and their products at a systemic level.

## Materials and methods

Thirty-five patients were selected at the start of the study. However, after the first round of selection, 15 who did not meet the inclusion crtiteria expressed later were excluded.20 participants (10 male and 10 female, with an age range of 45–50 years; mean age 47.1 years) were recruited from the outpatient unit of department of periodontology after obtaining their written informed consent. The study was approved by the Institutional Ethics Committee of Sri Ramachandra University (IEC-NI/10/MAY/16/14). The study was carried out in accordance with the Helsinki Declaration of 1975, as revised in 2000. Informed consent was obtained from all the participants and/or legal guardians for the study. Selection of the study participants was based on the following inclusion criteria: 1) a diagnosis of chronic generalized periodontitis as defined by the 1999 report of the International Workshop for a Classification of Periodontal Diseases and Conditions [14] 2) Presence of all the permanent teeth excluding third molars, 3) Attachment loss of > 1 mm in more than 30% of sites examined. The exclusion criteria were: 1) smokers or smokeless tobacco users, 2) Alcoholics, 3) history of any systemic diseases or currently on any medications, 4) pregnant or lactating women, 5) individuals who had taken any anti-inflammatory medication 1 week prior to enrolment in the study, 6) history of periodontal treatment in the past 6 months. A periodontal probe was used to record clinical parameters such as (a) presence or absence of plaque %, (b) positive or negative BOP elicited within 15 seconds of probing (c) probing depth (PD) recoded at 6 sides of the teeth measured from the gingival margin to the gingival sulcus in mm (d) clinical attachment level (CAL) measured from cementoenamel junction to the gingival sulcus recorded in mm. The gingival index was used to record the gingival inflammation.

### Sample collection

At baseline, whole peripheral blood samples were collected in two separate EDTA coated vacutainers by a trained phlebotomist using a sterile aseptic protocol. One sample was subjected for processing and monocyte subsets were determined using flow cytometry and another for analysis of CRP level by high performance nephalometry. Complete full-mouth scaling and root planing (non-surgical periodontal therapy) was performed in a single visit according to a previously published protocol [15]. Three weeks after the periodontal treatment, peripheral blood samples were collected again as previously mentioned above to determine monocyte subsets and CRP levels. The clinical recorder was blind to the treatment parameters (both pre and post).

### Protocol for flow cytometry analyses

Specific markers for identification of monocyte subsets such as fluorochrome tagged monoclonal antihuman antibodies,§ CD14 phycoerythrin (PE) (obtained from hybridization mouse Sp2/0 myeloma cells with spleen cells from BALB/c mice immunized with peripheral blood monocytes from a patient with rheumatoid arthritis, CD16 fluorescein isothiocyanate (FITC) (3G8 FITC mouse anti-human antibody.), and human leukocyte antigen- DR (HLA-DR) allophycocyanin (APC) (G46–6 allophycocyanin (APC) mouse anti-human antibody) were used in the present study. The reagents used for flowcytometric analysis were the specific lysis solution, Phosphate buffered saline (pH 7.4), wash buffer (PBS with 10% fetal calf serum [FCS]), and 1% paraformaldehyde [16].

### Protocol for direct immunofluorescent staining of whole peripheral blood samples

Samples of whole blood were stained by direct immunofluorescence following the previously documented protocol [12]. Briefly to 200 μL of blood sample taken in a tube addition of 7 μL CD14 (PE), 10 μL CD16 (FITC), and 5 μL HLA-DR (APC) was done and mixed slowly. Incubation in a dark room at a temperature of 20 °C to 25 °C was done for thirty minutes following which 2 mL addition of 1x FACS lysing solution was done. Following ten-minute incubation in dark at room temperature of 20 °C to 25 °C approximately centrifugation at 500 x g for 5 minutes and supernatant was discarded. To this addition of 3 mL PBS with 10% FBS (wash buffer) was done and centrifuged at 500 x g for 5 minutes. The supernatant was discarded and addition of 0.5 mL 1% paraformaldehyde solution was done and mixed thoroughly. The cells were acquired in a flow cytometer, analyzed using flow cytometry analysis software.

### Statistical analysis

The collected data were analyzed using IBM SPSS Statistics software version 23.0. Descriptive statistics, including mean, median, interquartile range (IQR), and standard deviation (SD), were calculated to summarize the central tendency and spread of the data. To assess the effect of non-surgical periodontal therapy on CD14 + CD16+ monocyte counts, we estimated the mean or median differences between pre-treatment (baseline) and post-treatment measurements. For normally distributed data, we calculated the mean difference and its associated 95% confidence interval (CI). For data with a markedly skewed distribution, we determined the median difference and its 95% CI, as suggested by the reviewer. To investigate the significance of the observed differences between paired groups (before and after treatment), we utilized the Wilcoxon signed-rank test for non-normally distributed data. The results of this analysis were visually represented using Whisker’s Box plots to display the distribution of data and highlight any outliers or trends. Additionally, to assess the relationship between non-classical monocyte counts and C-reactive protein (CRP) levels, we employed Pearson’s correlation analysis. This allowed us to examine potential associations between these variables. Throughout the statistical analyses, a significance level of α = 0.05 was used to determine statistical significance.

## Results

The clinical periodontal indices of the study population evaluated at baseline and 3 weeks post treatment are summarized in Table 1.

**Table 1:** Clinical Parameters of the Study Population

Parameter	Baseline	3 weeks post-operative	***p*** value
Plaque Index (%)	86 ± 6^a^	13 ± 5^a^	<0.01
Bleeding on Probing (%)	67 ± 8^a^	7 ± 4^a^	<0.01
Gingival Index	2.01 ± 0.53^a^	0.48 ± 0.34^a^	<0.01
Probing depth (mm)	6.45 ± 1.15^a^	3.35 ± 0.87^a^	<0.01
CAL (mm)	3.45 ± 0.99^a^	2.60 ± 0.88^a^	<0.01
CRP (mg/L)	3.03 ± 0.64^a^	1.54 ± 0.22^a^	<0.01

^a^values expressed as mean ± SD

### Effect of non-surgical treatment of periodontal parameters and CRP

The periodontal parameters evaluated at baseline and 3 weeks post non-surgical treatment were plaque %, BOP, gingival index, probing depth and clinical attachment loss (Table 1). There was a statistically significant improvement in all the clinical parameters at 3 weeks post treatment compared to baseline (*p* < 0.01, Wilcoxon signed rank test). The mean CRP level at baseline was 3.03 + 0.64 mg/L which reduced to 1.54 + 0.22 mg/L 3 weeks post non-surgical treatment (*p* < 0.01, Wilcoxon signed rank test).

### Levels of monocyte subsets in peripheral blood before and after non-surgical treatment

**Fig. 1:**
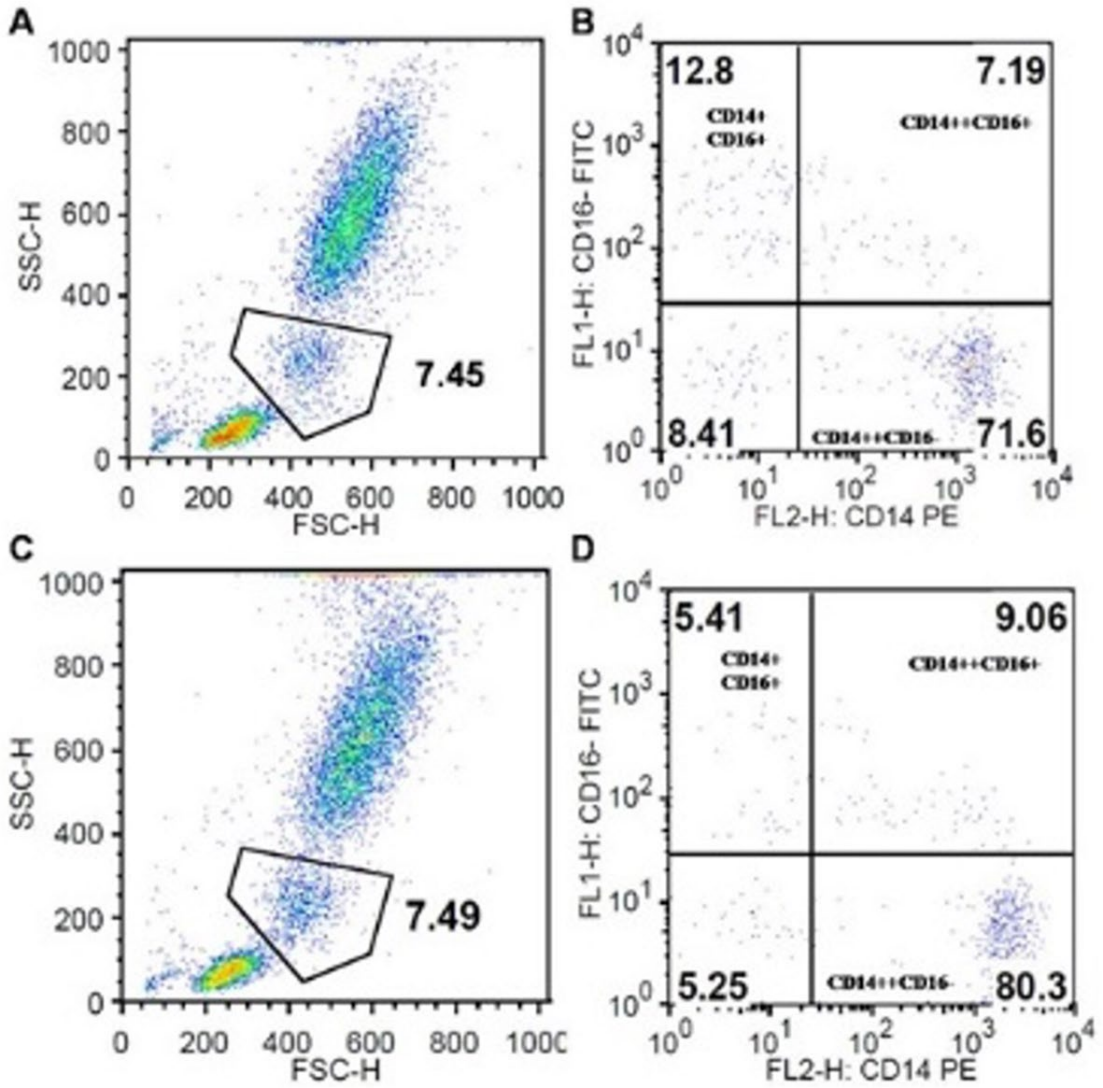
Effect of nonsurgical periodontal treatment on the proportions of monocyte subsets in peripheral blood. **A** Gating of monocytes in peripheral blood at baseline, **B** Monocyte subsets in peripheral blood at, lower right quadrant (CD14++CD16-classical monocytes), Upper right quadrant (CD14++CD16+ intermediate monocytes), upper left quadrant (CD14 + CD16+ non-classical monocytes), **C** Gating of monocytes in peripheral blood 3 weeks post non-surgical treatment, **D** Monocyte subsets in peripheral blood 3 weeks post non-surgical treatment, lower right quadrant (CD14++CD16-classical monocytes), Upper right quadrant (CD14++CD16+ intermediate monocytes), upper left quadrant (CD14 + CD16+ non-classical monocytes)

In the processed blood samples, the monocytes were gated as a separate region based on the forward and sideward scatter (Fig. 1 a, c). These gated monocytes were further analysed based on the expression of CD14 and CD16 to identify the subset of monocytes. Three distinct subpopulations were recognized lower right quadrant (CD14++CD16-classical monocytes), Upper right quadrant (CD14++CD16+ intermediate monocytes), upperleftquadrant (CD14 + CD16+ non-classical monocytes) (Fig. 1 b, d).

**Fig. 2:**
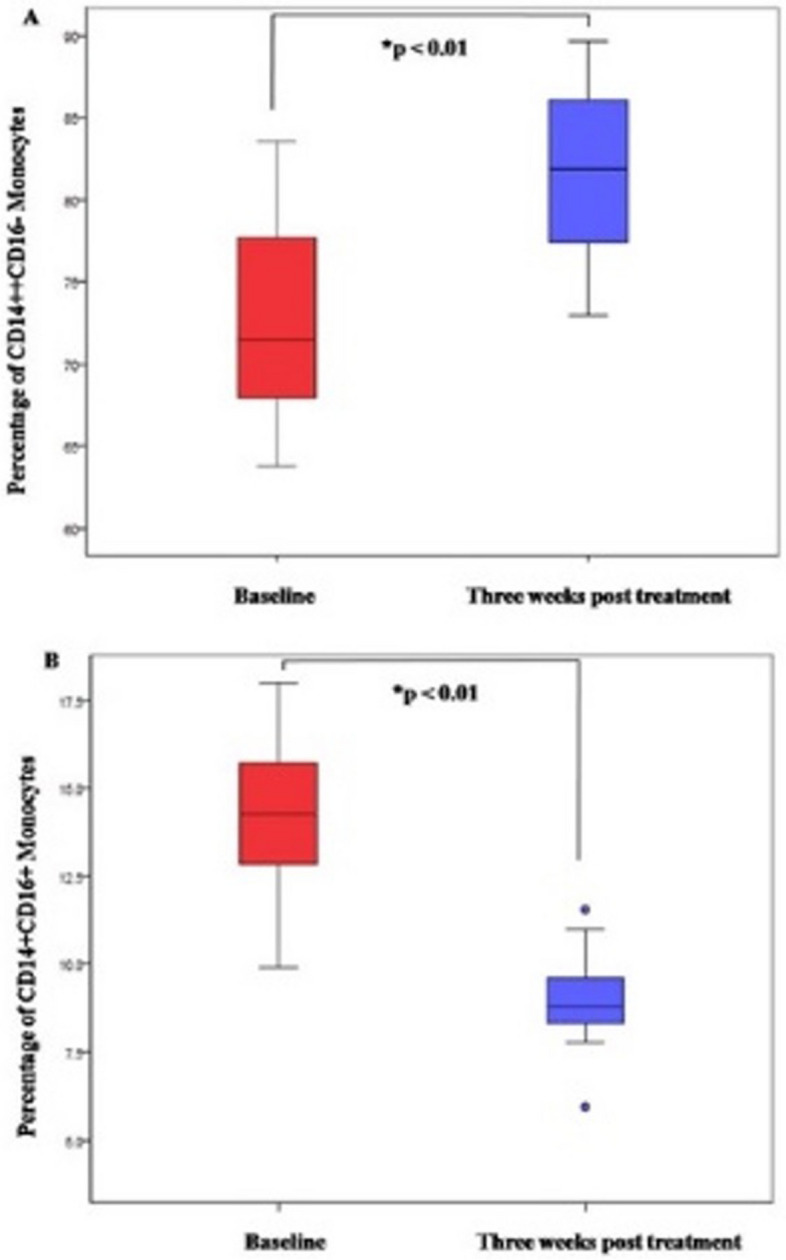
**A** Box plot representation of the percentage of classic monocytes in peripheral blood of at baseline and 3 weeks post treatment. *Wilcoxon signed rank test. **B** Box plot representation of the percentage of non-classic monocytes in peripheral blood of at baseline and 3 weeks post treatment. *Wilcoxon signed rank test

The results of the present study revealed a mean of 72 + 5% CD14++ CD16- classical monocytes in the peripheral blood at baseline. Three weeks after non-surgical periodontal treatment, the percentage of CD14++ CD16- classical monocytes increased to 81% + 5% (*P* < 0.01, Wilcoxon signed rank test) (Fig. 2 a).

The baseline mean percentage of CD14 + CD16+ non-classical monocytes in the peripheral blood at baseline was found to be 13% + 2%. Three weeks after non-surgical periodontal treatment a reduction in the percentage of CD14 + CD16+ non-classical monocytes to 8% + 1% was observed (*P* < 0.01, Wilcoxon signed rank test) (Fig. 2 b).

A distinct reduction in the percentage of non-classical monocytes and a concomitant increase in classical monocytes were observed in the peripheral blood samples 3 weeks following non-surgical periodontal treatment compared to baseline.

### Correlation between CD14 + CD16+ non-classical monocytes and CRP

**Fig. 3:**
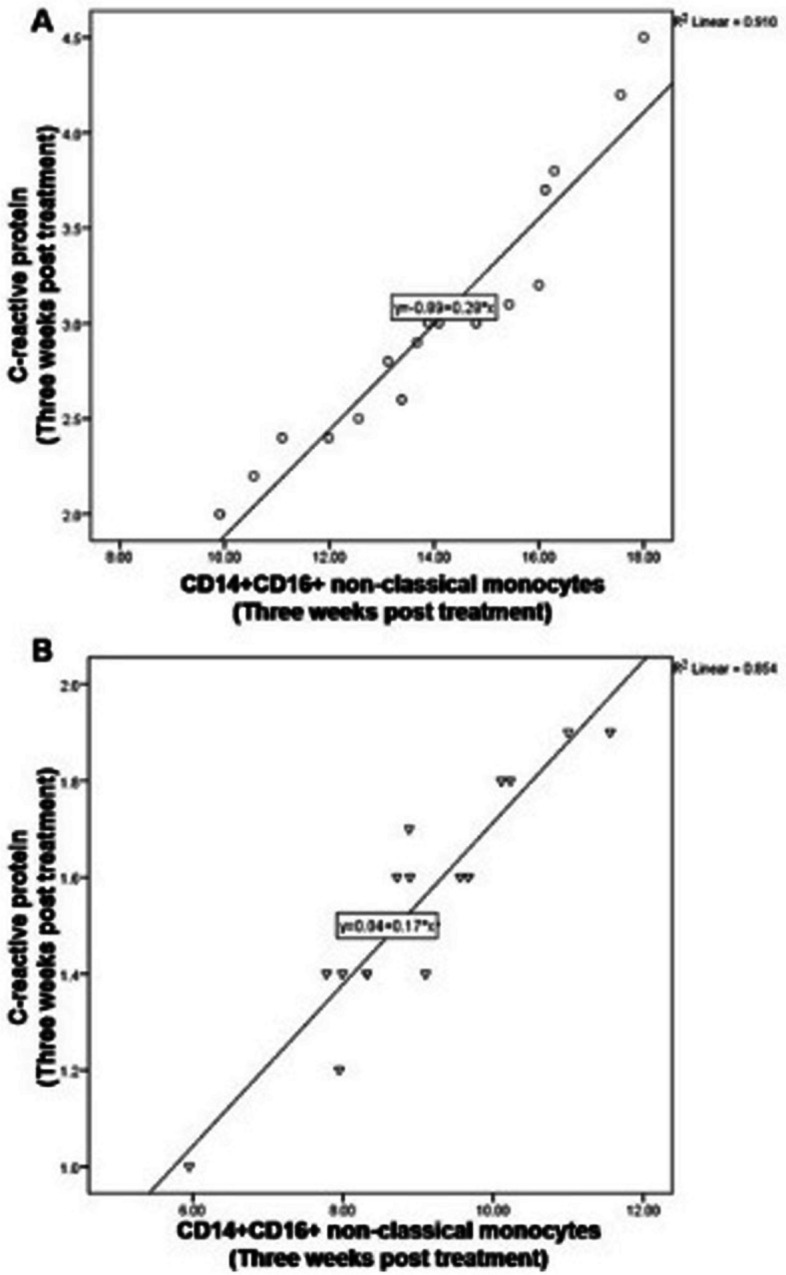
CRP value showed a significant positive correlation with the percentage of CD14 + CD16+ non-classical monocytes at **A**) baseline, **B**) 3 weeks post treatment

The correlation between CRP values and CD14 + CD16+ non-classical monocytes was analyzed using Pearson’s correlation. CRP value showed a statistically significant positive correlation with the percentage of CD14 + CD16+ non-classical monocytes at baseline (R^2^ = 0.910, *p* < 0.01, Pearson’s Correlation test, Fig. 3 a) and 3 weeks after non-surgical periodontal treatment (R^2^ = 0.854, *p* < 0.01, Pearson’s Correlation test, Fig. 3 b).

In table 2, descriptive statistics were calculated for CD14 + CD16+ monocyte counts both before and after the non-surgical periodontal therapy. At baseline, the mean monocyte count was 120.5, which significantly decreased to 85.3 post-treatment, resulting in a substantial mean difference of 35.2 (95% CI: 27.8 to 42.6). Similarly, the median monocyte count exhibited a noteworthy decline from 118.0 to 88.0, reflecting a median difference of 30.0 (95% CI: 25.1 to 34.9). The interquartile range (IQR) reduced by 6.5 (95% CI: 4.2 to 8.8) from 25.0 at baseline to 18.5 post-treatment. These statistics collectively indicate a significant reduction in CD14 + CD16+ monocyte counts following the treatment, supported by the consistency of these findings as denoted by the 95% confidence intervals. The standard deviation (SD) also decreased from 12.3 to 9.7, illustrating a decrease in data variability.

**Table 2:** Descriptive Statistics for CD14 + CD16+ Monocyte Counts; *p* value< 0.05 with statistical significance

Statistic	Pre-Treatment (Baseline)	Post-Treatment	Mean/Median Difference	95% Confidence Interval
**Mean**	120.5	85.3	35.2	(27.8, 42.6)
**Median**	118.0	88.0	30.0	(25.1, 34.9)
**IQR**	25.0	18.5	6.5	(4.2, 8.8)
**SD**	12.3	9.7	–	–

Table 3 presents the results of statistical analyses conducted to assess the significance of changes in CD14 + CD16+ monocyte counts and their relationship with C-reactive protein (CRP) levels. In the analysis comparing pre-treatment and post-treatment monocyte counts using the Wilcoxon signed-rank test, the test statistic is not specified but is indicated as significant (*p*-value < 0.05, α = 0.05). This suggests that the non-surgical periodontal therapy had a significant impact on CD14 + CD16+ monocyte counts. Furthermore, the correlation analysis conducted between CD14 + CD16+ monocyte counts and CRP levels using Pearson’s correlation coefficient revealed a positive correlation with a coefficient (r) of 0.42. This positive association was statistically significant, as indicated by the p-value of less than 0.05 (α = 0.05). Therefore, changes in CD14 + CD16+ monocyte counts were found to be related to changes in CRP levels.

**Table 3:** Statistical Analysis for CD14 + CD16+ Monocyte Counts and Correlation with CRP

Analysis	Test Statistic or Coefficient	***p***-value	Significance Level (α)
**Pre-Treatment vs. Post-Treatment (Wilcoxon Signed-Rank Test)**	–	<0.05	0.05
**CD14 + CD16+ Monocytes vs. CRP (Pearson’s Correlation)**	0.42	<0.05	0.05

## Discussion

The present clinical intervention study demonstrates the efficacy of non-surgical periodontal treatment on alteration of the monocyte subsets in the peripheral blood. It has already been documented that there is an elevation in the non-classical monocyte and monocyte derived macrophages in the blood and gingival tissues of chronic periodontitis patients compared to age and gender matched healthy subjects [12]. This finding is of paramount importance in modulating the pathogenesis of periodontitis. The non-classical subsets of monocytes are known to produce aberrantly higher amounts of pro inflammatory cytokines such as IL 1 beta and TNF alpha when challenged with bacterial LPS [10]. It is well known that the localized microbial burden in the periodontium is high in chronic periodontitis due to the sustained maintenance, survival and growth of periodontal pathogens in the plaque biofilm which serves as an ecological niche [17, 18]. It should also be noted that many periodontal pathogens such as *Porphyromonasgingivalis* [19] and *Aggregatibacteractinomycetemcommitans* [20] are tissue invaders. In this scenario the role of the periodontal pathogens in stimulating the non-classical monocytes to produce higher amounts of cytokines cannot be underrated. Equally pivotal is the role played by the periodontal microenvironment in chronic periodontitis characterized by high concentrations of cytokines and inflammatory mediators [21–25]. A study has researched the role played by epigenetic phenomena in increasing the proportions of non-classical monocyte subsets [26]. It can be understood that chronic periodontitis could be regarded as an epigenetic regulator of monocyte subset selection and phenotype maintenance which explains the findings of the present study with regard to elevated non classical monocyte in blood samples compared to their classical counterparts at baseline. A significant finding of the present study is the reduction in non-classic monocytes and increase in classic monocytes which was accompanied by an improvement of all clinical periodontal parameters including CRP levels encountered following non-surgical periodontal therapy compared to baseline measures [27]. This non-surgical periodontal treatment performed, consisted of scaling and root planning performed together in the same visit. It has been documented that scaling and root planning are the cornerstones of periodontal therapy and form a part of phase 1 or ateotrophic phase of periodontal treatment [28]. This treatment aims to remove supra and subgingival plaque and calculus and smoothens the root surfaces relieving them of root bound endotoxin [29]. The present study results on improvement of periodontal parameters following scaling and root planning are in accordance with studies that have demonstrated the efficacy of scaling and root planning on reduction of plaque scores, gingival bleeding and clinical resolution of gingival inflammation. Based on published literature which has demonstrated significant reduction of periodontal parameters post 3 weeks after scaling and root planing we used the same 3 week window in our study to assess improvement of clinical parameters and monocyte counts [30]. But so far no study has demonstrated the reduction of non-classical monocytes subsets in peripheral blood and a concomitant increase of classical monocytes following scaling and root planning. The present study results are in accordance with a previous study that has demonstrated reversal of non-classical to classical monocytes in tuberculosis patients following anti tuberculosis therapy [8]. The intriguing finding in the present study is the fact that the reversal of monocyte subsets has occurred without the use of any topical or systemic pharmacological agents. This can be justified as despite development of a plethora of pharmacological agents for periodontal disease management, periodontal disease resolution still relies on well performed removal of plaque and calculus which are considered the prime aetiological factors in initiating and perpetuating periodontal disease [31–33]. Also, the properties of the plaque biofilm such as the presence of drug metabolizing enzymes and the quick development of bacterial antibiotic resistance [34, 35] still explains why scaling and root planning are of colossal importance in periodontal disease management. Another important finding of the present study is the significant reduction of CRP levels in blood following periodontal therapy which can be significantly correlated with the monocyte subsets. This finding highlights the systemic effects of periodontal therapy in reducing inflammatory burden in terms of markers such as CRP, an important acute phase reactant involved in the pathobiology of periodontitis in addition to many other systemic diseases. Classical monocytes demonstrate antimicrobial activity by increased expression of myeloperoxidase (MPO), lysozyme C precursor (LYZ), S100 calcium binding protein A9 (S100A9), eosinophil cationic protein precursor (RNase3), phospholipase B domain containing 1 (PLBD1), and Cathepsin G (CTSG), at both mRNA and protein levels [36]. Expression of pro-inflammatory mediators, particularly, S100A12, S100A9, and S100A8 is a cardinal feature of this subset, but other stimuli can potentially mediate even tissue repair functions, such as wound healing, angiogenesis, and coagulation which are critical functions that are required for periodontal tissue healing [7, 37, 38]. The systemic effect of elevated classical monocytes following periodontal non-surgical therapy is another important finding as this could have an impact on other organ systems of the body. Periodontal disease has already been recognized as a significant risk factor for several systemic diseases like diabetes mellitus [39, 40], rheumatoid arthritis [41, 42], atherosclerosis [43, 44] and adverse pregnancy outcomes [45, 46]. It is well known that non classical monocytes play a major role in mediating tissue destruction in many systemic conditions [47]. Given the fact that periodontal non-surgical therapy could reduce the numbers of non-classical monocytes it could also reduce the risk of systemic disease and complications caused by preexisting periodontal disease.

In conclusion the study findings reiterate the positive influence of non-surgical periodontal therapy in reduction of non-classical monocyte subsets and a parallel elevation of classical monocytes blood. The study results reflecting post treatment levels of CD14+ CD16+ monocytes is comparable with CD14+ CD16+ monocyte counts of systemically and periodontally healthy individuals [12]. Future studies of this kind with increased sample size and elaborate investigation into markers of periodontal and systemic inflammation are required in patients with chronic periodontitis and comorbid systemic disease. If positive results are obtained, it could be elucidated that cost effective non-surgical periodontal therapy still remains the most important phase of periodontal therapy and should be well performed and followed up with effective surgical therapy where necessary.

## Conclusion

The non-surgical periodontal treatment results in a significant reduction in non-classical monocyte counts and CRP levels in the blood. Thus, periodontal treatment potentially modulates host response effectively and this may contribute to halting the progression of tissue destruction.

## Data Availability

Dr. Raghunanthan Jagannathan will have access to the data that were the basis for this article,and can be reached out for data in case is needed for review.
